# Evaluation of models determined by neutron diffraction and proposed improvements to their validation and deposition

**DOI:** 10.1107/S2059798318004588

**Published:** 2018-07-24

**Authors:** Dorothee Liebschner, Pavel V. Afonine, Nigel W. Moriarty, Paul Langan, Paul D. Adams

**Affiliations:** a Molecular Biophysics and Integrated Bioimaging Division, Lawrence Berkeley National Laboratory, Berkeley, CA 94720, USA; b Department of Physics and International Centre for Quantum and Molecular Structures, Shanghai University, Shanghai 200444, People’s Republic of China; c Neutron Science Directorate, Oak Ridge National Laboratory, PO Box 2008, Oak Ridge, TN 37831, USA; d Department of Bioengineering, University of California, Berkeley, CA 94720, USA

**Keywords:** model validation, neutron crystallography, PDB data mining, H/D exchange, *Phenix*

## Abstract

The Protein Data Bank (PDB) contains a growing number of models that have been determined using neutron diffraction or a hybrid method that combines X-ray and neutron diffraction. The advantage of neutron diffraction experiments is that the positions of all atoms can be determined, including H atoms, which are hardly detectable by X-ray diffraction. This allows the determination of protonation states and the assignment of H atoms to water molecules. Because neutrons are scattered differently by hydrogen and its isotope deuterium, neutron diffraction in combination with H/D exchange can provide information on accessibility, dynamics and chemical lability. In this study, the deposited data, models and model-to-data fit for all PDB entries that used neutron diffraction as the source of experimental data have been analysed. In many cases, the reported *R*_work_ and *R*_free_ values were not reproducible. In such cases, the model and data files were analysed to identify the reasons for this mismatch. The issues responsible for the discrepancies are summarized and explained. The analysis unveiled limitations to the annotation, deposition and validation of models and data, and a lack of community-wide accepted standards for the description of neutron models and data, as well as deficiencies in current model refinement tools. Most of the issues identified concern the handling of H atoms. Since the primary use of neutron macromolecular crystallography is to locate and directly visualize H atoms, it is important to address these issues, so that the deposited neutron models allow the retrieval of the maximum amount of information with the smallest effort of manual intervention. A path forward to improving the annotation, validation and deposition of neutron models and hybrid X-ray and neutron models is suggested.

## Abstract 2

Models of crystal structures determined by neutron diffraction and deposited in the Protein Data Bank to date were analysed. The lessons learned from this data-mining effort are summarized and suggestions for improvements to the deposition and validation of neutron models are outlined.

## 1. Introduction   

**Figure 1:**
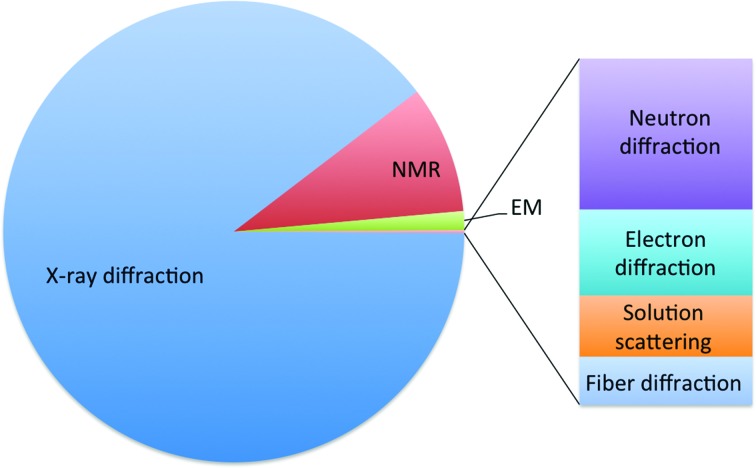
Experimental methods used to determine models in the PDB. The pre-dominant method is X-ray diffraction, followed by NMR and cryo-EM. Other methods are shown in the bar chart.

The predominant method to determine the three-dimensional structure of macromolecules is X-ray crystallography (Fig. 1 ▸), which is based on the interaction between X-rays and the electrons of the atoms constituting the crystal. Neutron diffraction is a complementary technique that relies on the interaction of neutrons with atomic nuclei. The neutron scattering cross-section, which determines the probability of a neutron being scattered by a nucleus, varies by element (or isotope) in a nonlinear fashion, as opposed to X-rays, where the scattering increases with the number of electrons. This is why neutron diffraction complements X-ray diffraction by enabling the location of very light atoms or ions such as hydrogen or protons in protein structures. As the knowledge of H-atom positions is important for determining the proton­ation states and reaction pathways of proteins (Engler *et al.*, 2003 ▸; Weber *et al.*, 2013 ▸; Haupt *et al.*, 2014 ▸; Casadei *et al.*, 2014 ▸; Howard *et al.*, 2016 ▸), neutron diffraction is able to provide valuable information for the understanding of catalytic mechanisms and ligand binding (Yamaguchi *et al.*, 2009 ▸; Bryan *et al.*, 2013 ▸; Knihtila *et al.*, 2015 ▸).

However, neutron diffraction may be challenging in practice for the following reasons.

**Figure 2:**
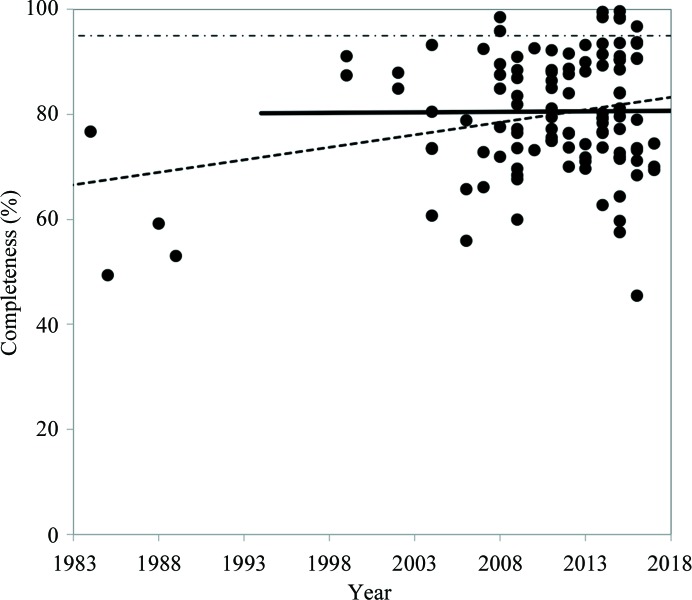
Completeness of neutron data per year. Reported resolution and σ cutoffs were applied. The dashed–dotted horizontal line indicates 95% completeness. The dashed and solid lines represent linear least-squares fits using all data and the data from 1999 to 2017, respectively. The latter fit (solid line) shows that the average completeness has not changed significantly during the past 25 years.

(i) *Experimental*. The beam flux at neutron sources is relatively weak compared with X-ray sources, necessitating the use of larger crystals (typically at least 0.1 mm^3^) and longer data-collection times for the experiment (Howard *et al.*, 2011 ▸; Weber *et al.*, 2013 ▸; Ng *et al.*, 2015 ▸). To date, the smallest crystal used for neutron data collection had a volume of 0.05 mm^3^ (Howard *et al.*, 2016 ▸). It is difficult to grow crystals of most proteins to such large sizes, and the number of proteins that can be explored by neutron crystallography is therefore relatively small. Furthermore, data-collection times are typically several days to a month on contemporary neutron sources (Blakeley *et al.*, 2008 ▸; Coates *et al.*, 2015 ▸; Chen & Unkefer, 2017 ▸). As hydrogen has an incoherent scattering cross-section that contributes to a high neutron scattering background level, it is preferable to replace hydrogen by deuterium, which has a much smaller incoherent scattering cross-section. Further, deuterium has a larger coherent scattering cross-section than hydrogen, and replacing hydrogen by deuterium therefore increases the signal-to-background ratio of diffraction peaks. Accessible H atoms in polar bonds (such as N—H, S—H and O—H, known as exchangeable H atoms or labile sites) can be either fully or partially exchanged for deuterium by soaking crystals in a deuterated buffer for several days prior to the diffraction experiment. However, in order to replace hydrogen by deuterium in nonpolar covalent bonds (such as C—H, known as non-exchangeable H atoms or nonlabile sites) protein expression must take place using fully deuterated reagents to produce what are referred to as perdeuterated samples. Obtaining perdeuterated samples can be a costly and time-consuming process (Price & Fernandez-Alonso, 2017 ▸) and can be an experimental obstacle. Apart from lowering the background scattering, perdeuterated samples offer several other benefits, such as the ability to use smaller crystal volumes, higher resolution data and faster data collection. Many studies are therefore performed with perdeuterated crystals (Meilleur *et al.*, 2013 ▸; Coates *et al.*, 2014 ▸; Cuypers *et al.*, 2016 ▸; Li *et al.*, 2017 ▸; Shu *et al.*, 2000 ▸; Fisher *et al.*, 2014 ▸; Blakeley *et al.*, 2015 ▸).
(ii) *Quality of the diffraction data*. Neutron data typically have a lower completeness compared with X-ray data. It is desirable that the completeness of a typical X-ray data set is greater than 95% (Dauter, 2017 ▸), but only a few neutron data sets satisfy this criterion (Fig. 2 ▸). The majority of data sets are less complete, averaging about 80%, owing to several factors including the relatively low flux of available neutron beams, reduction in signal to noise owing to incoherent scattering if any hydrogen is present, and the limited data-collection time available on highly oversubscribed macromolecular neutron crystallo­graphy instruments (for example, the oversubscription rate on the MaNDi and IMAGINE beamlines at the Spallation Neutron Source and High Flux Reactor neutron sources at Oak Ridge National Laboratory is typically greater than 300%). We observe that the completeness of neutron data has not improved notably during the past 25 years.
(iii) *Model building and refinement*. Using neutron diffraction data, H (or D) atoms can be refined individually along with the non-H atoms. If this strategy is applied, the number of parameters to be refined increases substantially, as about half of the atoms in a protein are H atoms. Furthermore, the neutron scattering length of hydrogen is negative, which can lead to scattering cancellation in medium- to low-resolution nuclear scattering length density maps when hydrogen is bound to atoms with a positive scattering length, such as in CH_2_ groups.^1^ To avoid negative scattering of H atoms, hydrogen can be partially or fully exchanged by using soaked or perdeuterated crystals (see above). However, the presence of different levels of H/D exchange makes model building more complicated, as there can be both H and D atoms, or either of them, at one location. We note that if the occupancy ratio of the H and D atoms at exchanged sites is about 0.6:0.4 the scattering is canceled [for illustrations, see Afonine *et al.* (2010 ▸) and references therein]. To tackle challenges in the model refinement process owing to low data completeness, low signal to noise and the increased number of parameters, the concept of joint X-ray and neutron refinement (hereafter referred to as joint XN refinement) was introduced (Coppens, 1967 ▸; Orpen *et al.*, 1978 ▸; Wlodawer, 1980 ▸; Wlodawer & Hendrickson, 1982 ▸; Adams *et al.*, 2009 ▸; Afonine *et al.*, 2010 ▸). In joint XN refinement a single model is simultaneously refined against X-ray and neutron data. Both data sets should be collected at the same temperature and should ideally be from the same or a highly isomorphous crystal, although this cannot always be realized. Patched versions of programs originally designed for the refinement of X-ray structures were made available to perform refinement using neutron data (Ostermann *et al.*, 2002 ▸; Engler *et al.*, 2003 ▸; Kurihara *et al.*, 2004 ▸). Also, as the number of neutron structures is still rather small, there are as yet no community-wide conventions for dealing with models obtained from joint refinement and/or that contain both H and D atoms.

When computational tools are developed, it is desirable to exercise the new algorithms using all available data and models (see, for example, Afonine *et al.*, 2009 ▸; Weichenberger *et al.*, 2015 ▸). This ensures that the new developments work not only on the developer’s favourite examples but are also robust enough to work generally, which is the key for automated software development. New tools for joint XN refinement are being developed in the framework of the *PHENIX* software suite (Adams *et al.*, 2010 ▸). To test the algorithms, all neutron models and diffraction data available as of 8 September 2017 in the Protein Data Bank (PDB; Berman *et al.*, 1977 ▸, 2000 ▸) were analyzed. An approach for the early detection of issues that could cause problems is to use the deposited data and model to calculate *R*_work_ and *R*_free_, and compare the obtained values with the published values. A mismatch may be indicative of various issues, ranging from trivial typos to incomplete or incorrect annotations in the deposited data. We find a surprising number of models that show large differences (reaching up to 30%) between the reported and recomputed *R* factors. These models and data were inspected in order to determine the origin of the differences. This study summarizes the lessons learned from the data-mining effort.

## 2. Materials and methods   

### 2.1. Collecting the data from the PDB   

All computations were performed with *PHENIX* tools (Adams *et al.*, 2010 ▸). Models determined by neutron diffraction were identified using the ‘experimental method’ search option on the PDB website. The model PDB and data files were obtained with the *phenix.fetch_pdb* tool. Information relevant to recomputing *R* factors using the same conditions as were used for refinement of the final structure by the depositors were automatically extracted from the PDB file header: minimum and maximum resolution limits and σ cutoff as well as the twin law, if present. Furthermore, crystallo­graphic *R* factors (*R*_work_ and *R*_free_), the deposition year and the program used for refinement were obtained from the PDB file header.

### 2.2. Diffraction data labels for joint XN data sets   

In the case of models determined by joint XN refinement, the corresponding data file should contain at least two data arrays: one for the neutron data and one for the X-ray data. It is therefore important to know which data array corresponds to which experiment. In the data CIF file the item _diffrn.details can be used to describe the details of the diffraction measurement, such as ‘first data set reflections X-ray diffraction’ and ‘second data set reflections neutron diffraction’. We note that annotations could not be parsed automatically. The keyword or sentence was not consistently the same and in several instances only one data array had an annotation while the other did not. However, a practical way to determine which data array corresponds to which experiment is to compute *R*_work_ using X-ray and neutron scattering factors for both data arrays; the wrong set of scattering factors leads to higher *R* factors.

### 2.3. Model files   

#### 2.3.1. Assessment of hydrogenation state   

We define the hydrogenation state as a model feature describing how the experimentalists chose to model H-atom sites (using H, D or H and D). The presence of H and D atoms in the PDB file was used to sort models into four different categories.

(i) *Predominantly D atoms are present*. This case occurs for crystals of perdeuterated protein containing deuterated solvent.
(ii) *Predominantly H atoms are present*. This case occurs for crystals of hydrogenous protein containing hydrogenous solvent.
(iii) *Significant amounts of both H and D atoms are present, with more H atoms than D atoms*. This case occurs for crystals of hydrogenous proteins containing a relatively small amount of deuterated solvent, or for crystals of perdeuterated protein containing relatively large amounts of hydrogenous solvent.
(iv) *Significant amounts of both H and D atoms are present, with more D atoms than H atoms*. This case occurs for crystals of perdeuterated protein containing deuterated solvent, if metabolites were used during protein expression that were not fully deuterated, if some D atoms have been back-exchanged by H atoms during sample preparation or handling, or for crystals of hydrogenous proteins that contain relatively large amounts of deuterated solvent.

Case (i) is worthy of further consideration. Even if a protein is expressed from organisms cultured in deuterated reagents and crystallization is performed in deuterated solutions, there is a chance that the sample will have been exposed to ambient hydrogenated moisture at some stage. It is therefore unlikely that all H atoms (100%) are replaced by D atoms (an all-D refinement protocol might nevertheless be chosen, for example to increase the data-to-parameter ratio). Also, it may happen that some D atoms back-exchange to hydrogen if hydrogenated reagents are used in one of the protein crystal-production steps (such as purification; Haupt *et al.*, 2014 ▸; Yee *et al.*, 2017 ▸). Some models therefore contain a majority of D atoms and very few H atoms. To prevent the misinterpretation of such a model as containing both H and D, which means that H atoms are at *all* exchangeable sites, a cutoff was applied. If more than 90% of atoms are of one type (H or D) this type is assigned. We chose 90% because it represents a compromise between a strict separation of perdeuterated *versus* hydrogenated and the experimental reality that even perdeuterated crystals can contain some H atoms.

Furthermore, for each model we determined the total number of H or D atoms and the number of H atoms, D atoms and exchanged sites in protein (or RNA/DNA) residues. Here, an exchanged site is not counted twice as belonging to the H and D atoms as well; for example, an H atom is either H, D or exchanged. A site was identified as being exchanged if both H and D were used to model it. The number of H or D atoms in other molecular species was also determined, including water molecules and ligands. Finally, the percentage of H/D-exchange sites per protein H and D atom was analysed.

#### 2.3.2. Properties of H and/or D atoms   

In addition to counting H and D atoms (§2.3.1), we also looked at (i) models containing H or D atoms with occupancies smaller than zero; (ii) models with incomplete *X*H_3_ groups (‘propeller groups’), *i.e.* if one H or D atom was missing; (iii) the use of standard *X*—H and *X*—D bond-length constraints; and (iv) the coordinates and atomic displacement parameters (ADPs) of corresponding H and D atoms in exchanged sites.

#### 2.3.3. Generation of H and/or D atoms   

If the deposited model did not contain H or D atoms according to the published information, they were generated using *phenix.ready_set*. If both H and D atoms were added at exchangeable sites, the occupancy ratio was set to 50:50. These curated models were used to test hypotheses about particular issues. The reported values in Tables 1 ▸, 2 ▸ and 3 ▸ are based on original models (unless curation was necessary to be able to process the file; for example, a few models contained corrupt atom names).

### 2.4. Model-to-data fit: computation of *R* factors   

To assess the model-to-data fit, *R*_work_ and *R*_free_ were computed using resolutions and σ cutoffs as reported in the PDB header or the literature. X-ray (Maslen *et al.*, 1992 ▸; Waasmaier & Kirfel, 1995 ▸; Grosse-Kunstleve *et al.*, 2004 ▸) and neutron (Sears, 1992 ▸) scattering tables were used as appropriate.

## 3. Results and discussion   

### 3.1. Overview of neutron models deposited to date   

**Figure 3:**
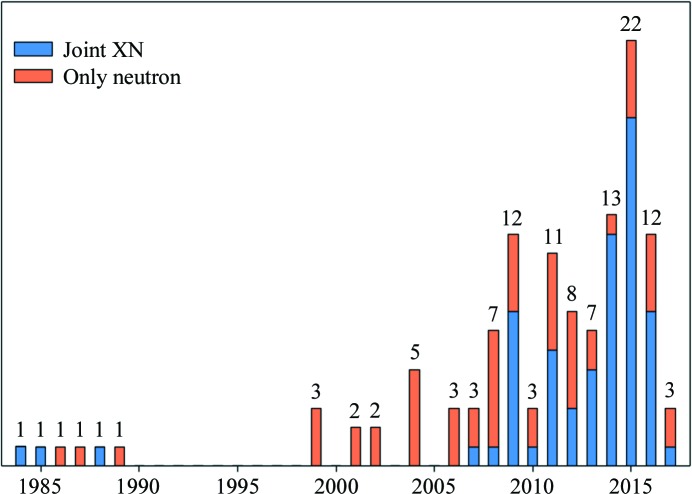
Cumulative number of neutron models in the PDB. Coral, models determined using neutron data alone. Blue, models determined using joint XN refinement.

As of 8 September 2017, the number of neutron diffraction models deposited in the PDB was 122. Fig. 3 ▸ shows the cumulative number of neutron models per year. The first model in the database was determined in 1984 and corresponds to the structure of a bovine pancreatic trypsin inhibitor determined by joint XN refinement (PDB entry 5PTi; Wlodawer *et al.*, 1984 ▸). However, several structural reports predate the establishment of the PDB, such as a model of myoglobin (Schoenborn, 1969 ▸), or were not deposited in the PDB, such as a model of crambin (Teeter & Kossiakoff, 1984 ▸).

It can be noted that no models were deposited between 1990 and 1998 owing to the unavailability of macromolecular neutron crystallography facilities in the early 1990s. The reactors at the Institut Laue–Langevin (ILL) in Grenoble and the High Flux Beam Reactor (HFBR) at Brookhaven were unavailable from 1990 to 1995 and from 1989 to 1991, respectively (Chen & Unkefer, 2017 ▸). Also, some neutron structures were not deposited in the PDB, such as a model of concanavalin A (Habash *et al.*, 1997 ▸). Several factors have changed this situation and have recently increased the rate of model deposition. New and advanced neutron sources have begun operation, including the SNS in the USA, the FRM-2 reactor in Germany and J-PARC in Japan. Additional macromolecular neutron crystallo­graphy beamlines have been built, including LADI (Cipriani *et al.*, 1997 ▸) in France; PCS (Langan *et al.*, 2004 ▸), MaNDi (Coates *et al.*, 2015 ▸) and IMAGINE (Meilleur *et al.*, 2013 ▸) in the USA; BioDiff (Ostermann & Schrader, 2015 ▸) in Germany; and iBIX (Kurihara, Tanaka, Muslih *et al.*, 2004 ▸) in Japan. New methods and technologies have been developed, such as the development of the neutron image-plate detector (Niimura *et al.*, 1994 ▸) and the development of new types of macromolecular neutron crystallography beamlines based on the use of powerful time-of-flight techniques at spallation sources (Langan *et al.*, 2004 ▸). The rate of structure deposition will increase further with several next-generation advanced neutron sources that are under construction or commissioning, including the ESS in Sweden (https://europeanspallationsource.se) and the CSNS in China (http://english.ihep.cas.cn/csns/).

The total number of deposited structures has grown since the 1980s, but the number of depositions per year is low compared with X-ray crystallography and has varied between three and 22 during the past decade. Among the 122 deposited structures, 55 were determined using neutron data alone (coral in Fig. 3 ▸) and 67 were obtained from joint XN refinement (blue in Fig. 3 ▸). Most of the recently deposited structures were refined using the joint XN refinement method. The development of robust refinement algorithms for joint XN refinement has enabled the increased use of macromolecular neutron crystallography and has provided more complete (including all atoms) and more accurate structures.

**Figure 4:**
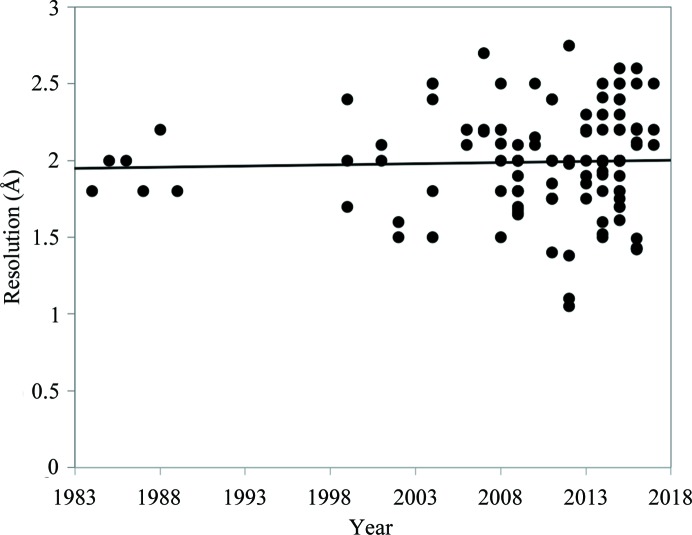
Resolution of neutron models in Å as a function of the year of deposition (black circles). The black line represents a linear fit. The average resolution did not improve over a period of more than 35 years, as shown by the linear fit, which is almost parallel to the *y* axis at a resolution of 2 Å.

Fig. 4 ▸ shows the resolution of the neutron diffraction data sets as a function of deposition year. Interestingly, the average resolution has not improved in a period of more than 35 years, with the majority of data sets having resolutions of between 1.5 and 2.5 Å. The mean data resolution for all 122 deposited models is 1.99 Å. The highest resolution was reported for PDB entry 4AR3 (Cuypers *et al.*, 2013 ▸), which has neutron data extending to 1.05 Å resolution. This is related to the primary reason that researchers conduct neutron crystallography studies of biological macromolecules. Neutron crystallography is not used to determine the structures of biological macromolecules; that is best performed using X-ray crystallography. Rather, neutron crystallography addresses critical science questions that require the direct location and visualization of functionally important H atoms or protons. Using neutron crystallography, H atoms can be located at resolutions of 2.5 Å or less, *i.e.* the resolution of almost all deposited neutron structures. An exception is PDB entry 3VXF, which was determined with neutron data collected to 2.75 Å resolution (Yamada *et al.*, 2013 ▸).

The earlier models were refined with *PROLSQ* (Hendrickson & Konnert, 1979 ▸) and some models determined with neutron data alone were refined using *X-PLOR* (Brunger, 1992 ▸) or *SHELX* (Gruene *et al.*, 2014 ▸; Sheldrick, 2015 ▸). We note that the neutron community is increasingly using programs tailored to handle neutron data, such as *PHENIX* (Afonine *et al.*, 2010 ▸) and *nCNS* (Adams *et al.*, 2009 ▸), which can be used for joint XN refinement (Table 3 ▸).

### 3.2. Data files   

#### 3.2.1. Availability   

Six data sets from neutron diffraction experiments in the PDB do not have diffraction data at all (PDB entries 2XQZ, 1GKT, 1io5, 1LZN, 1NTP and 6RSA). Three joint XN data sets have only X-ray data (PDB entries 4CVJ, 3KYX and 5JPR), while in six cases only neutron data are available (PDB entries 3QF6, 4Q49, 3KKX, 5DPN, 3iNS and 5A93).^2^ In these cases it is possible to refine models against the neutron data alone, but the joint refinement cannot be reproduced. The absence of the X-ray data is largely a result of limitations in earlier PDB deposition processes. It is important that experimental data should be deposited and made available. Of the 122 models determined *via* neutron or joint XN refinement, nine do not have neutron data, which is more than 7%.

#### 3.2.2. Type of diffraction data   

When multiple data arrays associated with a PDB entry are available, it is important to be able to identify whether an array corresponds to X-ray or neutron data. Only 27 of the 67 joint data sets had an annotation in the CIF file, whereas a majority of 40 models did not have any specification. These annotations cannot be processed automatically as they are inconsistent or incomplete in many cases. For example, in some instances there was an annotation for only one array while the other array had none. By comparing *R* factors using X-ray and neutron scattering factors for both data arrays, their type could be identified. However, this may be complicated if this is convoluted with the issue of incorrect H/D assignment (see §3.3.2).

#### 3.2.3. Incomplete or missing cross-validation (*R*_free_) sets   

The *R*_free_ flags in 24 data sets do not match the available data. This means that at least one reflection in the data file did not have an *R*_free_ flag assigning it to the test set or the working set. If *R*_free_ flags are present, *PHENIX* tools require a data file to have these flags for every reflection.

#### 3.2.4. Wrong data annotations   

It is important to know whether diffraction data are intensities or amplitudes. For example, the neutron data array for PDB entry 2iNQ is indicated as structure-factor amplitudes in the CIF file. The recomputed *R*_work_ and *R*_free_ are 26.6 and 30.6%, respectively. If the data array is treated as intensities, *R*_work_ and *R*_free_ are 20.9 and 25.0%, respectively, which are much closer to the published values of 18.2 and 23.3%. This is likely to be owing to incorrect annotation during deposition or conversion.

### 3.3. Model files   

#### 3.3.1. Information in the PDB file header   

The information in the PDB file header can be incomplete, *i.e.* the values necessary to perform the refinement under the same conditions, such as the resolution limit or the σ cutoff, have not been included. Furthermore, there are cases where the information is different in the header and in the concomitant paper. For example, the header of PDB entry 1WQ2 reports 22.9 and 28.9% for *R*_work_ and *R*_free_, respectively, while the paper indicates values of 28.2 and 30.1% (Chatake, Mizuno *et al.*, 2003 ▸). The latter are similar to the recomputed *R* factors (28.5 and 31.1% for *R*_work_ and *R*_free_, respectively).

The H (or D) atoms and the presence of exchanged sites with both H and D are most likely to be the largest source of confusion in model files (discussed below).

#### 3.3.2. Availability of H/D atoms  

(i) *No H or D atoms are deposited*. One model (PDB entry 5KSC) was deposited without any H or D atoms on the protein residues (in contrast, all water molecules have two D atoms). The primary purpose of a neutron diffraction experiment is to obtain information about H atoms. If the deposited model is lacking H atoms, an important interpretation of the experimental result is not accessible.
(ii) *Wrong atom type is deposited*. PDB entry 1CQ2 contains H atoms in the protein chain, while all water molecules are D_2_O molecules. The PDB header suggests that the protein is fully deuterated (OTHER_DETAILS: PROTEIN IS FULLY DEUTERATED). Switching from the original to a fully deuterated model decreases *R*_work_ and *R*_free_ from 47.1 and 47.7% to 21.7 and 26.8%, respectively.
(iii) *Only one atom type at exchanged sites*. PDB entry 1C57 only contains H atoms, while the literature (Habash *et al.*, 2000 ▸) describes that the model was refined with D atoms at the backbone amide groups. Another model, an earlier version of which was in the PDB at the time this manuscript was prepared, contained H atoms with full occupancy, while no D atoms were present (the model was meanwhile curated and contains now both H and D). The issue of missing atoms at exchanged sites is difficult to detect, but is mainly associated with early structures that were determined while robust refinement methods were still being developed.
(iv) *Missing H or D atoms*. We found that several models with some lysine side-chain terminal NH_3_ groups or CH_3_ methyl groups did not contain all H or D atoms, *i.e.* one or two of the three H (or D) atoms were missing. For example, eight models contained at least ten residues where exactly one H atom was missing in propeller groups. When two (or one) of the atoms are present in the *X*H_3_ group, the location of other atoms is automatically determined. In some cases, the omission may reflect a different protonation or charge state, which may be functionally important. In fact, one of the goals of a neutron diffraction study may be to determine the charge state of a catalytically important lysine residue. In other cases, it is possible that the software unintentionally omitted the H atoms. To be able to distinguish these two scenarios, it should be explicitly marked when residues are in a charged state, such as for neutral lysine (for example, using a PRB remark). As H and D atoms are non-negligible scatterers in neutron models, their unjustified systematic omission deteriorates the model quality.

#### 3.3.3. Modeling of partially exchanged sites   

**Figure 5:**
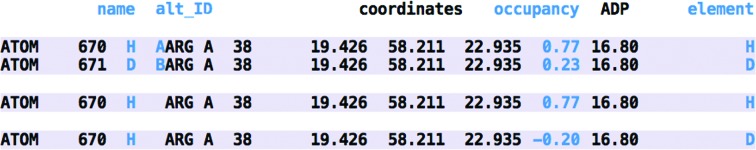
Illustration of PDB format for an exchanged amide H atom in an arginine residue (residue 38 of chain *A*). All three possibilities describe the same configuration: one H atom with occupancy *q*_H_ = 0.77 and a D atom with complementary occupancy *q*_D_ = 1 − *q*_H_ = 0.23. Top, method (i), using double conformation and different atom names. Middle, method (ii), implying the D atom with *q*_D_ = 1 − *q*_H_. Bottom, method (iii), using an apparent occupancy. The occupancy mimics the total scattering contributions of the H and D atoms, which are of different signs, and varies from −0.56 (H fully occupied) to 1 (D fully occupied).

Atomic models of partially deuterated crystals contain sites with both H and D atoms sharing the same location. This situation arises if only a fraction of a particular H atom of all of the molecules in the crystal was replaced by a D atom. At least three approaches for the simultaneous modeling of an H and D atom at the same location were found in models deposited in the PDB. Fig. 5 ▸ shows the PDB format for an amide H atom for the three modeling options. The PDB format lines describe the same information, *i.e.* an H atom with occupancy 0.77 and a D atom with occupancy 0.23 at the same location. The lines look rather different for the different methods and they are explained below.

(i) *Modeling the H and D atoms as a double conformation*. This is the most common approach, which aims to prevent the application of nonbonded repulsion restraints during refinement between the H and the D atoms. An important difference when compared with alternative conformations for an entire residue is that the atom names of the H and D atoms are different. For example, in the serine residue hydroxyl group, the H atom will be modeled in conformation *A* with the name ‘HG’, while the D atoms is in conformation *B* with the name ‘DG’. This method has both the H atom and the D atom in the model file. However, it is unclear how residues in alternate conformations and simultaneously containing exchanged H/D atoms will be defined. PDB entry 3VXF, for example, has alternative conformations of a leucine residue in conformations *A* and *C*, while the exchanged amide D atom has conformation *B*. This is the major limitation of this method. We note that 13 models had at least one H/D pair with erroneous atom names and had to be curated. For example, the amide H/D atoms H and D both had the alternative conformer identifier (ID) *A* in Ile10 of PDB entry 2VS2. The correction included changing the alternative conformer ID of one of the atoms to *B*.
(ii) *Only one atom type (H or D) is present in the PDB file with occupancy *q**. The other atom type is not present in the file, but it is situated at the same position as its exchanged partner atom and has a complementary occupancy of 1 − *q*. This scenario occurs in PDB entry 3BYC, which was determined from a soaked crystal, and should therefore contain both H and D atoms. The deposited model file only contains protein H atoms with occupancy *q*. A remark in the PDB header states that the D-atom occupancies are 1 − *q*. Other models have similar configurations but do not contain a remark (such as PDB entry 4G0C). An obvious disadvantage of this approach is that the deposited model is incomplete because it does not contain all H/D atoms and therefore such a model needs manipulation (adding missing D atoms) before it can be used. Although this is straightforward to interpret individually, it can lead to confusion during automatic data mining.
(iii) *Altering the definition of occupancy*. The occupancy of an atom reflects the fraction of molecules in the crystal in which this atom occupies a certain position. Therefore, to be meaningful the occupancy value is expected to be between zero and one. However, some PDB entries contain D atoms with negative occupancies (PDB entries 3CWH, 3KCJ, 3KCL, 3KCo, 3KKX, 3KMF and 3oTJ) in order to represent the H/D-exchange ratio. The value of the occupancy ranges from −0.56 (H fully occupied) to 1 (D fully occupied) (Kawamura *et al.*, 2011 ▸). In this approach the definition of the occupancy value is misused, as it does not reflect the occupancy of the atom in question. We note that of the seven models that use the apparent occupancy only one contains a remark explaining the modified meaning of the occupancy in the PDB file header (3oTJ). Furthermore, similarly to the second method, this approach yields an incomplete model, as not all H or D atoms are present in the file.

Clearly, all three of the above approaches have their particular advantages and limitations. Method (iii) correctly reflects the scattering factors during refinement but it creates occupancy definition issues for automatic PDB mining. Methods (ii) and (iii) lead to atom-incomplete models that require curation to be usable.

#### 3.3.4. Hydrogenation state   

**Figure 6:**
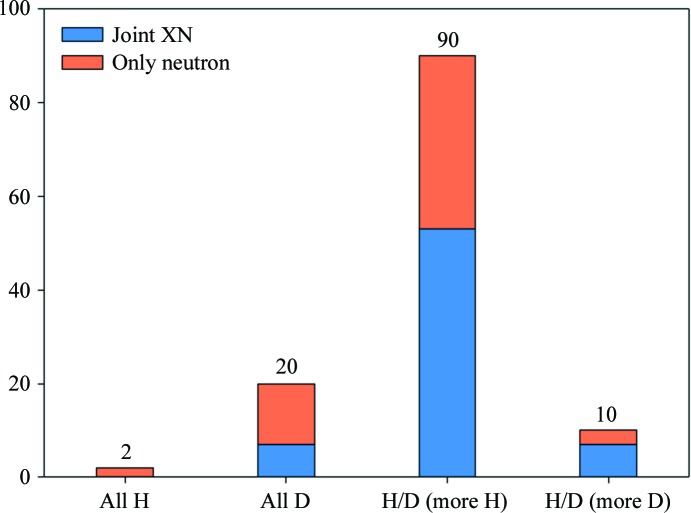
Distribution of hydrogenation states. The majority of models contain both H and D atoms.

Fig. 6 ▸ shows a histogram of the hydrogenation state, as determined by the procedure described in §2.3.1. Most models (88) contain significant amounts of both H and D atoms, with a majority of H atoms. It is likely that these models correspond to crystals of hydrogenated protein soaked in D_2_O. 19 models contain predominantly D atoms (among H and D) and are likely to originate from crystals of perdeuterated protein containing deuterated solvent. Ten models contain significant amounts of both H and D atoms, with a majority of D atoms. In most of these cases the proteins were expressed in a deuterated medium that contained D_2_O but with hydrogenated glycerol, which leads to mixed H/D occupancy at every nonexchangeable C—H site (PDB entries 4JEC, 5E5J, 5E5K and 5T8H), hydrogen labeling (PDB entry 3KYY; Gardberg *et al.*, 2010 ▸) or selective protonation or deuteration (Fisher *et al.*, 2014 ▸; PDB entry 4NY6). Five models are in the fourth category and contain mainly H atoms (among H and D), such as PDB entries 5D97 (a hydrogenated crystal) and 1NTP (contains a small number of exchanged H atoms).

As the hydrogenation state is difficult to assess algo­rithmically, we suggest that the PDB or mmCIF file should contain a specific keyword identifying the protonation state. For example: ‘protonation: H’ (or ‘D’ or ‘H and D’ in the other cases).

Table 1 ▸ shows a more detailed breakdown of the H- and D-atom count, sorted according to the hydrogenation state of the model, for protein residues, water molecules and other entities (such as ligands). The percentage of H/D sites represents how many of the total H sites in a protein are modeled with both an H and a D atom. A large number of models containing both H and D atoms do not have shared sites, *i.e.* a site is either occupied by an H atom or a D atom. Most notably, of the 88 models that contain more H than D atoms, 23 do not have any shared sites. It is not possible to determine algorithmically whether this choice was made on purpose (for example to decrease the number of refinable parameters by avoiding H/D-occupancy refinement) or whether the complementary atom is assumed to be accounted for but is not physically present in the file [such as for method (ii) described in §3.3.3].

For models containing shared sites, the ratio of exchanged sites and all modeled protein H atoms in the model in question varies between 4 and 23%. Notable exceptions are models 4JEC, 5E5J, 5E5K and 5T8H, where the ratio of exchanged H/D is 83, 78, 75 and 69%, respectively. As mentioned above, the samples for these models were prepared in a special manner and are expected to contain H and D atoms at the majority of sites (exchangeable and not exchangeable).

**Figure 7:**
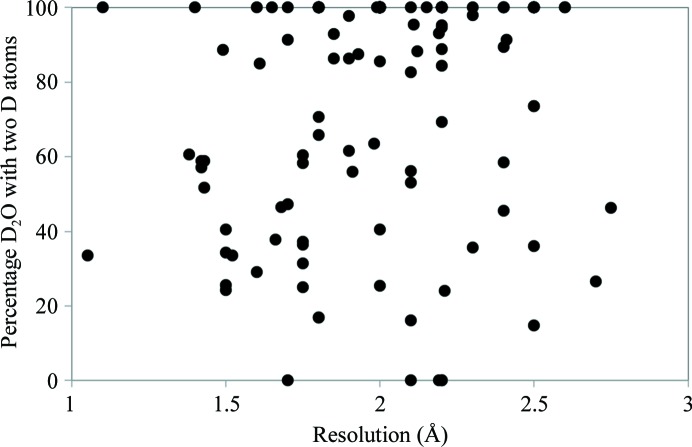
Percentage of water molecules with two D atoms as a function of neutron data resolution (black circles).

The table also lists the number of water molecules modeled with no, one or two D atoms. In 52 of the 122 models all water molecules were modeled as D_2_O molecules. However, it was reported that only a fraction of water molecules show a distinguished triangular shape in nuclear scattering length density maps that allows the location of both D atoms (Chatake, Ostermann *et al.*, 2003 ▸). Fig. 7 ▸ shows the percentage of water molecules modeled as D_2_O as a function of resolution. Only with the higher resolution data sets is it possible to accurately differentiate between different water species (OD^−^, D_2_O and D_3_O^+^).

#### 3.3.5. Properties of exchanged sites   

As D has a larger mass than H, it is expected that D has a lower ADP. However, the resolution of most macromolecular neutron diffraction data sets is not sufficient to detect this difference. Imposing the same ADPs and coordinates for H and D atoms is therefore a reasonable approximation. The sum of occupancies at H/D sites is constrained to 1. We analysed whether exchanged sites in all models fulfil these criteria.

Out of 81 models with at least one exchanged H/D, 20 have different coordinates (25%), ten have sites with different ADPs and eight and six have the sum of occupancies smaller and larger than one, respectively. The number of mismatches per model can range from one (one coordinate mismatch, PDB entry 3U2J) to 542 (coordinate mismatch and occupancy sum < 1; PDB entry 2DXM).

In some cases, the mismatch comes from model errors, such as in PDB entry 4JEC, where the HG3 atom of proline 1 (chain *A*) has the wrong atom name, which should be correctly indicated as HG2. It has the same coordinates and ADP as DG2 and the sum of occupancies *q*_DG2_ + *q*_HG3(HG2)_ = 1. DG3, on the other hand, is modeled as being fully occupied. It therefore cannot have an exchanged partner. 364 atoms suffer from mislabeled atom names in this model.

In other models, such as 3FHP, the H and D atoms of the amide N atom are modeled systematically with different coordinates. The distance between the atoms ranges from 0.01 Å (Gly20, chain *D*) to 0.5 Å (Leu6, chain *B*).

Model 3HGN has 232 sites with different ADPs (but the same coordinates) for the H and the D atom. The difference can reach up to 11 Å^2^ (Asn148, chain *A*). It is possible that the ADPs were refined individually for both atoms (as opposed to being constrained to be equal to each other, as is desirable).

#### 3.3.6. The covalent *X*—H bond lengths are set to standard X-ray distances   

**Figure 8:**
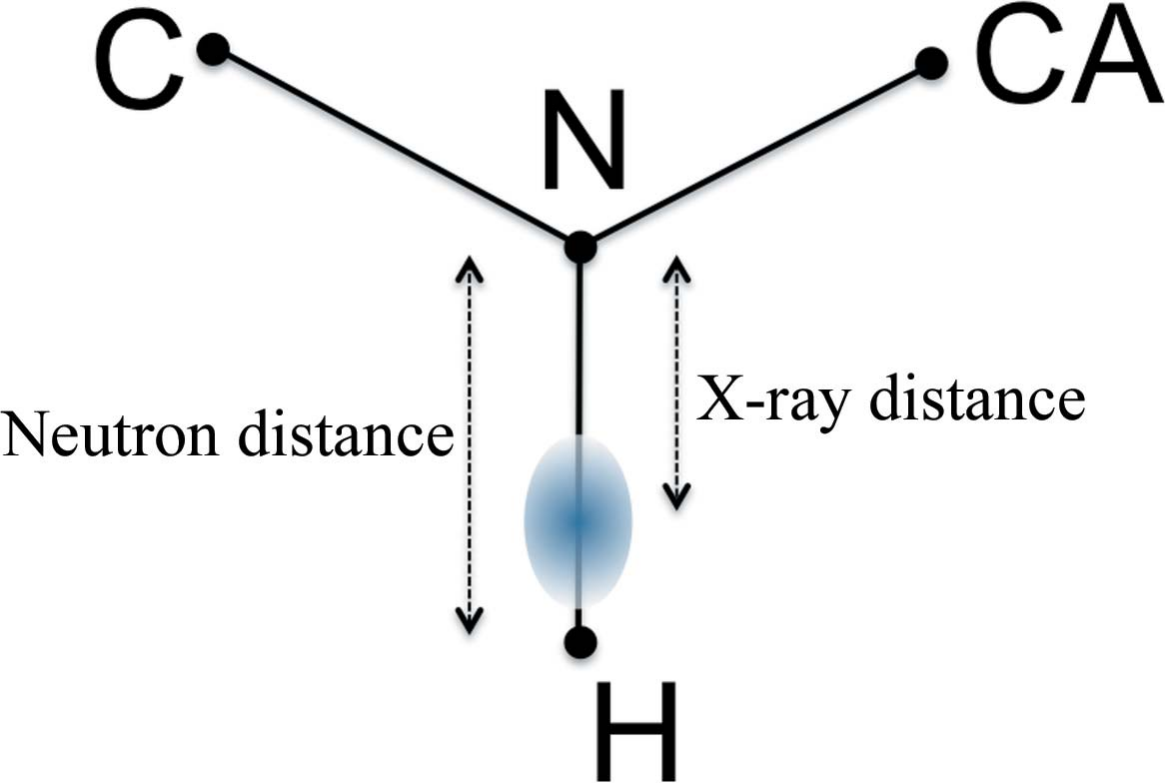
Schematic figure illustrating the X-ray and neutron N—H bond lengths for an amide H atom. The nuclei are represented by black spheres and the electron cloud of the H atom is represented by the blue gradient-coloured oval. The centre of the electron distribution is shifted towards the N atom along the N—H covalent bond.

The *X*—H bond length is different in models derived from X-ray and neutron diffraction data. X-rays interact with electrons, and in the case of the H atom (which has only one valence electron and therefore no core electrons) the electron distribution is shifted along the covalent *X*—H bond towards atom *X*. Neutrons interact with the nuclei, which are not affected by deformations of the valence electron density owing to chemical bonds. H-atom nuclear scattering length density peaks are therefore at a different location to electron-density peaks (Fig. 8 ▸), and *X*—H bond lengths thus appear to be shorter in X-ray models than in neutron models. The difference in bond length is 10–20% (Allen, 1986 ▸; Allen & Bruno, 2010 ▸), requiring that standard neutron distances be used for the refinement of H and D atoms in neutron models. It was mentioned by Gruene *et al.* (2014 ▸) that several neutron models were refined with X-ray *X*—H bond lengths. Of the 122 neutron models deposited in the PDB, the H (or D) atoms are located at X-ray distances in more than 40 models.

Using shorter (X-ray) instead of longer (neutron) bond lengths may not affect *R*-factor values greatly and the effect may largely depend on the data resolution (lower impact at lower resolution, greater impact at higher resolution). For example, in PDB entry 2GVE, which contains 4195 H atoms, most of them are placed at standard X-ray distances; the recomputed *R*_work_ and *R*_free_ are 24.7 and 30.0%, respectively. Using standard neutron distances, *R*_free_ decreases to 29.8% while *R*_work_ remains the same. However, to obtain a model that reflects the experimental data correctly, the *X*—H distances should be according to commonly accepted targets for neutron distances.

## 4. Summary of the lessons learned from the survey   

Table 2 ▸ provides a summary of the following parameters for all neutron models: PDB code, deposition year, H/D state, refinement program, high-resolution and σ cutoff, published and recomputed *R*_work_ and *R*_free_. Table 3 ▸ lists the same information for models from joint XN refinement, along with relevant cutoffs and *R* factors for the X-ray data sets.

To address the differences between models described in §3, we suggest that the following guidelines are adopted during the deposition and validation of neutron models.

(i) All H (D) atoms used in refinement should be deposited.
(ii) Information describing the experiment or the results should be correct and be consistent with the concomitant publication, such as resolution limits and σ cutoffs.
(iii) For joint models, all data should be made available, *i.e.* X-ray and neutron diffraction data, and the data arrays should be unambiguously marked.
(iv) A community-wide accepted description of H/D-exchanged locations did not exist during the early days of macromolecular neutron crystallography, and three different approaches have been used. Moving forward, there is an opportunity to adopt a new description that is compatible between different software packages and does not change the usual definition of existing parameters (such as the occupancy). As the community transitions to the mmCIF format,^3^ this is a good opportunity to address this issue. A solution could be more-than-one-letter alternative conformation IDs.
(v) We also note that when models determined *via* neutron diffraction are being deposited a validation report is generated but it is not made available in the PDB.^4^
(vi) There is a need for validation tools specifically designed for neutron crystallography. Current validation software either ignores H atoms or only uses them to validate heavy-atom positions and geometry.

It should be also noted that the PDB allows authors to correct a structure at any point, *i.e.* deposit a revised version, which could be an opportunity to curate some of the issues that are described in this report.

## 5. Development of a validation tool for H atoms   

The work described in this report led to the development of a new tool in *PHENIX* that can comprehensively validate neutron models and data. It is available in *PHENIX* release 1.13 and later. The following validation tasks are performed.

(i) Identification of missing H (or D) atoms.
(ii) An accounting of the number of H, D and exchanged H/D sites.
(iii) Identification of H/D sites with an occupancy ratio that leads to nearly full cancellation of their density (approximately 0.35/0.65). If such a site has a degree of freedom, it should be checked.
(iv) Identification of H/D sites with different coordinates, ADPs and unlikely occupancy values.
(v) A count of water molecules with zero, one or two D atoms.
(vi) A warning message if X-ray *X*—H distances are used.

Broader use of this tool will help address some of the issues that are raised in our analysis.

## 6. Conclusions   

Neutron models constitute a small fraction of the models deposited in the PDB; however, the information that they provide is unique and of great importance for understanding biological function. At present, X-ray crystallography is the method of choice for determining the structure of biological macromolecules. Neutron crystallography is used only in cases where a critical science question requires the direct localization and visualization of H atoms or protons. The initial goal of surveying neutron models was to verify the suitability of their use in the development and benchmarking of new robust computational tools for neutron crystallography. However, a preliminary assessment of model-to-data fit quality has revealed opportunities to improve the PDB annotation and validation methods and the deposition process itself. Implementation of the suggested improvements will minimize inconsistencies between the deposited neutron models available in the PDB and therefore the possibility of misinterpretation. Most of the issues identified concerned the handling of H and D atoms. The survey led to the development of a new tool in *PHENIX* that can comprehensively validate H and D atoms in protein models. Since the primary use of macromolecular crystallography is to locate and directly visualize H atoms, it is important to address these issues, so that deposited neutron models allow the retrieval of the maximum amount of information with the smallest effort of manual intervention.

## Figures and Tables

**Table 1:** H and D atoms in protein residues, waters and other entities Water molecules in alternative conformations were not counted among the categories with zero, one or two D atoms. This result is not shown, so the sum is not always equal to the total number of water molecules.

		Total		Protein				Water				Other	
PDB code†	Resolution (Å)	H	D	H	D	H/D	Ratio	Total water molecules	0 × D	1 × D	2 × D	H	D
Predominantly H													
5D97	1.8	929	0	848	0	0	0	107	44	45	18	0	0
1NTP	1.8	1440	154	1433	154	0	0	0	0	0	0	7	0
1XQN	2.5	2089	0	1755	0	334	0	227	60	0	167	0	0
1CQ2	2.0	1277	138	1247	0	0	0	69	0	0	138	30	0
1C57	2.4	2051	0	1755	0	0	0	148	0	0	148	0	0
Predominantly D													
2R24	2.19	0	2552	0	2542	0	0	285	285	0	0	0	10
3KYX	1.68	0	418	0	382	0	0	28	5	10	13	0	0
3QF6	1.85	0	668	0	542	0	0	73	10	0	63	0	0
3RYG	1.75	5	395	0	346	5	1.4	36	13	2	21	0	0
3RZ6	1.75	5	379	0	346	5	1.4	35	20	2	13	0	0
3RZT	1.75	6	385	0	342	6	1.7	35	9	15	11	0	0
3SS2	1.75	5	385	0	346	5	1.4	33	11	10	12	0	0
4AR3	1.05	0	557	0	423	0	0	149	75	18	50	0	16
4AR4	1.38	45	590	0	397	45	10.2	104	30	8	63	0	14
4BD1	2.0	0	2238	0	1985	0	0	145	21	0	124	0	5
4C3Q	2.2	0	2212	0	1985	0	0	125	14	0	111	0	5
4K9F	1.75	9	455	0	362	9	2.4	58	9	14	35	0	0
4PVM	2.0	127	1842	6	1637	121	6.9	89	41	12	36	0	0
4PVN	2.3	100	1836	6	1664	94	5.3	84	36	18	30	0	0
5A90	1.7	0	1977	0	1977	0	0	317	317	0	0	0	0
5A93	2.2	0	2109	0	2109	0	0	238	238	0	0	0	0
5CE4	1.9	0	1455	0	1062	0	0	179	1	0	175	0	33
5KSC	2.1	0	48	0	0	0	0	29	5	0	24	0	0
2XQZ	2.1	0	1982	0	1982	0	0	205	205	0	0	0	0
H and D (more H)													
1iU6	1.6	336	88	290	15	46	13.1	31	15	5	9	0	0
1L2K	1.5	1143	243	966	47	147	12.7	74	47	9	18	30	4
1V9G	1.8	122	106	96	32	6	4.5	44	15	0	29	20	10
1VCX	1.5	348	97	301	14	47	13.0	37	16	6	15	0	0
1WQ2	2.4	786	274	681	79	105	12.1	99	54	0	45	0	0
1WQZ	2.5	188	96	188	42	0	0	27	0	0	27	0	0
2DXM	2.1	4116	995	3454	227	542	12.8	201	88	0	113	120	0
2EFA	2.7	346	98	298	32	48	12.7	34	25	0	9	0	0
2GVE	2.2	2742	1453	2353	199	389	13.2	512	79	1	432	0	0
2iNQ	2.2	2147	595	2111	303	0	0	152	8	0	144	36	4
2MB5	1.8	1004	475	974	277	0	0	89	0	0	89	30	20
2VS2	2.0	2303	911	1831	48	420	18.3	220	0	0	220	52	3
2WYX	2.1	1938	617	1491	0	447	23.1	160	75	0	85	0	0
2YZ4	2.2	1356	559	1356	399	0	0	84	4	0	80	0	0
2Zoi	1.5	806	235	687	62	113	13.1	73	38	10	25	6	0
2ZPP	2.5	342	85	294	27	48	13.0	34	29	0	5	0	0
2ZWB	1.8	849	367	722	148	127	12.7	65	19	0	46	0	0
2ZYE	1.9	1591	520	1359	143	192	11.3	143	50	5	88	40	4
3A1R	1.7	783	383	664	96	119	13.5	92	8	0	84	0	0
3BYC	2.2	2211	494	2211	2	0	0	246	0	0	246	0	0
3CWH	2.2	2244	1144	2244	680	0	0	227	0	0	227	0	10
3FHP	2.11	688	333	592	67	96	12.7	89	4	0	85	0	0
3HGN	1.65	1588	814	1329	199	232	13.2	190	0	0	190	27	3
3iNS	2.2	605	186	605	186	0	0	325	325	0	0	0	0
3KCJ	1.8	2257	1158	2257	681	0	0	237	0	0	237	0	3
3KCL	2.0	2252	1087	2252	677	0	0	199	0	0	199	0	12
3KCo	1.8	2252	1310	2252	681	0	0	309	0	0	309	0	11
3KKX	2.0	1563	902	1563	448	0	0	227	0	0	227	0	0
3KMF	2.0	3579	1531	3459	933	0	0	299	0	0	299	120	0
3L45	1.8	782	338	641	15	141	17.7	91	0	0	91	0	0
3oTJ	2.15	1533	736	1533	496	0	0	120	0	0	120	0	0
3Q3L	2.5	8043	1726	6499	6	1544	19.2	238	150	0	86	0	0
3QZA	2.0	2730	1159	2305	205	425	14.5	264	0	0	264	0	1
3R98	2.4	2271	862	1728	1	513	22.9	174	0	0	174	30	0
3R99	2.4	2271	862	1728	1	513	22.9	174	0	0	174	30	0
3TMJ	2.0	1555	829	1555	447	0	0	191	0	0	191	0	0
3U2J	2.0	1698	389	1342	0	356	21.0	55	36	5	14	0	0
3VXF	2.75	2044	654	1749	226	277	12.3	158	85	0	73	18	5
3X2o	1.5	1067	375	823	6	238	22.3	152	63	50	39	6	3
3X2P	1.52	1017	380	759	3	209	21.5	149	43	56	50	49	12
4CVi	2.41	1991	827	1764	268	197	8.8	198	17	0	181	30	0
4CVJ	2.5	2078	870	1825	183	223	10.0	315	83	0	232	30	0
4DVo	2.0	2748	1216	2304	182	444	15.2	288	0	0	288	0	14
4FC1	1.1	289	147	252	26	37	11.7	42	0	0	42	0	0
4G0C	2.0	1561	741	1558	451	0	0	144	0	0	144	3	2
4GPG	1.98	1597	613	1341	165	256	14.5	140	37	14	89	0	0
4LNC	2.19	2759	1155	2308	166	440	15.1	289	13	7	269	11	4
4N3M	1.9	1866	743	1774	358	89	4.0	169	21	2	146	3	2
4N9M	2.3	1753	897	1753	527	0	0	187	3	1	183	0	3
4PDJ	1.99	1265	511	962	0	262	21.4	119	0	0	119	41	11
4Q49	1.6	1563	828	1563	452	0	0	188	0	0	188	0	0
4QCD	1.93	2058	759	1634	11	395	19.4	185	0	23	162	29	6
4QDP	2.0	2753	1238	2320	183	427	14.6	312	0	0	312	6	4
4QDW	1.8	2721	1140	2307	217	405	13.8	259	0	0	259	9	0
4QXK	2.2	939	423	788	53	144	14.6	111	0	0	111	7	4
4RSG	1.91	1015	309	888	98	120	10.8	75	33	0	42	7	7
4S2D	2.0	1306	622	1034	59	260	19.2	151	0	0	151	12	1
4S2F	2.0	1258	593	1053	96	205	15.1	146	0	0	146	0	0
4S2G	2.0	1283	610	1038	69	245	18.1	148	0	0	148	0	0
4S2H	1.7	1300	668	1023	51	277	20.5	170	0	0	170	0	0
4XPV	2.0	1344	692	998	0	346	25.7	173	0	0	173	0	0
4Y0J	2.0	1563	735	1563	457	0	0	139	0	0	139	0	0
4ZZ4	1.8	806	242	687	42	119	14.0	107	44	45	18	0	0
5C6E	2.0	2118	705	1743	144	315	14.3	123	0	0	123	60	0
5C8i	2.2	1912	566	1596	102	309	15.4	77	0	0	77	7	1
5CCD	2.6	1753	454	1502	49	251	13.9	77	0	0	77	0	0
5CCE	2.5	1717	485	1569	201	137	7.2	70	0	0	70	11	7
5CG5	2.4	2745	736	2147	9	592	21.5	106	34	10	62	6	1
5CG6	2.4	2746	683	2142	7	589	21.5	47	3	2	42	15	1
5DPN	1.61	1365	332	1110	0	211	16.0	60	9	0	51	44	19
5EBJ	2.5	1544	481	1387	140	157	9.3	92	0	0	92	0	0
5GX9	1.49	889	365	771	105	112	11.3	80	7	0	71	6	0
5JPC	2.5	1745	431	1457	38	281	15.8	53	0	0	53	7	6
5JPR	2.2	1893	944	1467	0	396	21.3	390	112	8	270	30	0
5K1Z	2.6	1749	435	1462	44	271	15.3	58	0	0	58	16	4
5KWF	2.21	3785	1061	2884	1	870	23.2	258	138	58	62	31	8
5MNX	1.42	1420	536	1105	14	313	21.9	134	21	27	79	2	3
5MNY	1.43	1414	524	1093	1	320	22.6	129	13	35	76	1	3
5MoN	1.42	1484	593	1171	22	311	20.7	161	20	37	92	2	3
5Moo	1.43	1460	558	1141	13	318	21.6	149	21	41	77	1	3
5PTi	1.8	344	229	344	103	0	0	63	0	0	63	0	0
5RSA	2.0	693	472	693	216	0	0	128	0	0	128	0	0
5TKi	2.12	3035	1301	2670	284	317	9.7	382	39	0	337	48	14
5VG1	2.1	2031	915	1578	0	453	22.3	231	0	0	231	0	0
5WEY	2.5	1707	642	1405	68	288	16.4	139	0	0	139	14	8
1GKT	2.1	2077	370	2045	288	0	0	255	214	0	41	32	0
1LZN	1.7	695	497	695	267	0	0	243	128	0	115	0	0
6RSA	2.0	692	451	684	225	0	0	112	0	0	112	8	2
H and D (more D)													
3KYY	1.66	61	348	61	306	0	0	37	9	14	14	0	0
3QBA	1.4	117	122	99	34	0	0	41	0	0	41	18	6
4JEC	2.0	1398	1838	35	240	1332	82.9	131	0	0	131	31	4
4NY6	1.85	108	548	108	470	0	0	42	3	0	39	0	0
5Ai2	1.75	57	457	4	370	53	12.4	48	28	7	12	0	3
5E5J	2.0	1300	1860	5	362	1262	77.5	116	0	0	116	33	4
5E5K	2.3	1292	1805	41	373	1218	74.6	105	0	0	105	33	4
5T8H	2.2	1181	1854	28	480	1122	68.8	124	0	0	124	31	4
5VNQ	2.2	328	1409	0	1009	328	24.5	36	0	0	36	0	0
1io5	2.0	696	766	696	264	0	0	251	0	0	251	0	0

† For the PDB code naming convention used in this article, please see Moriarty (2015 ▸).

**Table 2:** Summary for models determined using neutron data alone The models are sorted according to their deposition year, except for the six models without data, which are at the end. Hydrogenation-state abbreviations: all_h, model contains predominantly H atoms; all_d, model contains predominantly D atoms; hd_and_h, both H and D present, with more H than D; hd_and_d, both H and D present, with more D than H.

						Published		Recomputed	
PDB code	Year	Hydrogenation state	Program	Resolution (Å)	σ Cutoff	*R*_work_(Å)	*R*_free_(Å)	*R*_work_(Å)	*R*_free_(Å)
2MB5	1989	hd_and_h	*PROLSQ*	1.8	n/a	11.2	n/a	23.4	24.0
1C57	1999	all_h	*X-PLOR*	2.4	0	27.0	30.1	29.8	33.4
1CQ2	1999	all_d	*X-PLOR*	2.0	0	16.0	25.0	47.1	47.7
1iU6	2002	hd_and_h	*CNS*	1.6	3	20.1	22.8	20.2	22.7
1L2K	2002	hd_and_h	*CNS*	1.5	0	20.1	23.8	19.8	23.4
1V9G	2004	hd_and_h	*CNS*	1.8	3	22.2	29.4	24.1	31.2
1VCX	2004	hd_and_h	*CNS*	1.5	2	18.6	21.7	18.5	21.2
1WQ2	2004	hd_and_h	*CNS*	2.4	1	28.2	30.1	28.5	31.1
1WQZ	2004	hd_and_h	*CNS*	2.5	1	28.4	32.6	27.8	33.1
1XQN	2004	all_h	*CNS*	2.5	2	26.6	32.0	35.1	35.3
2DXM	2006	hd_and_h	*CNS*	2.1	1	19.7	26.0	20.1	26.1
2GVE	2006	hd_and_h	*SHELX*	2.2	3	27.1	31.9	24.7	29.8
2iNQ	2006	hd_and_h	*SHELX*	2.2	3	18.2	23.3	20.9	25.0
2EFA	2007	hd_and_h	*CNS*	2.7	3	21.6	29.1	24.1	29.3
2YZ4	2007	hd_and_h	*CNS*	2.2	0	27.9	31.2	27.5	31.0
2VS2	2008	hd_and_h	*CNS*	2.0	0	21.9	28.1	22.9	22.6
2Zoi	2008	hd_and_h	*CNS*	1.5	1	19.2	21.9	19.0	21.5
2ZPP	2008	hd_and_h	*CNS*	2.5	1	22.1	26.0	22.9	27.7
2ZWB	2008	hd_and_h	*CNS*	1.8	0	22.3	24.7	22.4	24.5
3CWH	2008	hd_and_h	*SHELX*	2.2	0	23.7	28.8	27.0	25.7
3FHP	2008	hd_and_h	*CNS*	2.11	3	17.9	24.7	16.7	23.2
2WYX	2009	hd_and_h	*PHENIX*	2.1	1.52	22.3	25.8	22.3	25.9
2ZYE	2009	hd_and_h	*PHENIX*	1.9	n/a	19.3	22.2	19.5	22.4
3A1R	2009	hd_and_h	*CNS*	1.7	0	19.5	23.8	18.0	22.4
3KMF	2009	hd_and_h	*nCNS*	2.0	2.5	25.0	30.0	26.0	26.1
3Q3L	2010	hd_and_h	*PHENIX*	2.5	0.06	22.1	26.8	23.0	27.6
3RYG	2011	all_d	*PHENIX*	1.75	1.8	18.1	20.0	21.8	22.5
3RZ6	2011	all_d	*PHENIX*	1.75	1.56	20.8	23.8	24.2	24.1
3RZT	2011	all_d	*PHENIX*	1.75	1.53	20.2	24.9	24.3	25.7
3SS2	2011	all_d	*PHENIX*	1.75	1.53	21.0	24.2	24.3	25.7
3U2j	2011	hd_and_h	*PHENIX*	2.0	0	23.2	27.2	23.2	26.9
4AR3	2012	all_d	*PHENIX*	1.05	1.33	19.9	23.7	19.2	22.7
4AR4	2012	hd_and_d	*PHENIX*	1.38	0	18.6	22.6	16.9	21.4
4BD1	2012	all_d	*PHENIX*	2.0	0	22.0	25.7	21.2	24.6
4FC1	2012	hd_and_h	*SHELX*	1.1	0	21.1	25.3	20.8	25.3
4G0C	2012	hd_and_h	*nCNS*	2.0	n/a	26.7	28.3	26.9	25.8
4C3Q	2013	all_d	*PHENIX*	2.2	1.36	19.2	24.0	19.1	23.8
4K9F	2013	all_d	*PHENIX*	1.75	n/a	19.9	24.1	19.9	24.2
4RSG	2014	hd_and_h	*PHENIX*	1.91	1.41	24.9	28.7	25.5	28.9
4ZZ4	2015	hd_and_h	*PHENIX*	1.8	n/a	19.7	22.1	19.9	20.8
5A90	2015	all_d	*PHENIX*	1.7	1.33	19.2	22.7	19.4	22.7
5Ai2	2015	hd_and_d	*PHENIX*	1.75	1.34	23.31	28.64	25.2	29.3
5D97	2015	all_h	*PHENIX*	1.8	0	22.0	22.2	21.7	22.0
5GX9	2016	hd_and_h	*PHENIX*	1.49	1.39	15.8	20.0	15.9	20.0
5KSC	2016	all_d	*SHELX*	2.1	0	24.3	28.3	33.1	34.7
5MNX	2016	hd_and_h	*PHENIX*	1.42	1.35	16.6	20.6	16.7	20.7
5MNY	2016	hd_and_h	*PHENIX*	1.43	1.34	16.4	19.3	16.5	19.5
5VG1	2017	hd_and_h	*PHENIX*	2.1	2.38	18.7	26.5	18.9	26.6
5VNQ	2017	hd_and_d	*PHENIX*	2.2	1.43	24.2	28.0	24.4	28.2
Models without neutron diffraction data									
2XQZ	2010	all_d	*PHENIX*	2.1	1.55	22.5	25.9	n/a	n/a
1GKT	2001	hd_and_h	*SHELX*	2.1	0	23.5	27.4	n/a	n/a
1io5	2001	hd_and_d	*X-PLOR*	2.0	None	21.0	32.3	n/a	n/a
1LZN	1999	hd_and_h	*X-PLOR*	1.7	2	20.4	22.1	n/a	n/a
1NTP	1987	hd_and_h	Unknown	1.8	None	18.7	None	n/a	n/a
6RSA	1986	hd_and_h	*PROLSQ*	2.0	None	None	None	n/a	n/a

**Table 3:** Summary for models determined using joint XN refinement The models are sorted according to their deposition year.

				Neutron						X-ray					
						Published		Recomputed				Published		Recomputed	
PDB code	Year	Hydrogenation state	Program	Resolution (Å)	σ Cutoff	*R*_work_(Å)	*R*_free_(Å)	*R*_work_(Å)	*R*_free_(Å)	Resolution (Å)	σ Cutoff	*R*_work_(Å)	*R*_free_(Å)	*R*_work_(Å)	*R*_free_(Å)
5PTi	1984	hd_and_h	*PROLSQ*	1.8	n/a	21.7	n/a	17.8	19.9	0.94	n/a	21.8	n/a	18.9	19.5
5RSA	1985	hd_and_h	*PROLSQ*	2.0	3.0	18.3	n/a	17.7	19.3	2.0	3.0	15.9	n/a	16.1	17.1
3iNS	1988	hd_and_h	*PROLSQ*	2.2	n/a	19.1	n/a	18.0	18.6	1.5	n/a	18.2	n/a	n/a	n/a
2R24	2007	all_d	*PHENIX*	2.19	1.53	25.7	29.1	25.3	28.8	1.75	1.33	12.9	16.6	12.5	16.4
3BYC	2008	hd_and_h	*nCNS*	2.2	2.5	26.4	31.5	29.7	32.6	2.2	2.5	23.3	25.2	20.8	23.1
3HGN	2009	hd_and_h	*PHENIX*	1.65	n/a	19.6	21.6	19.6	21.6	1.2	n/a	14.9	16.3	14.5	15.6
3KCJ	2009	hd_and_h	*nCNS*	1.8	2.5	17.3	18.1	18.1	17.9	2.0	2.5	17.9	18.7	16.6	17.1
3KCL	2009	hd_and_h	*nCNS*	2.0	2.5	18.8	21.1	20.1	20.2	2.0	2.5	17.3	19.4	16.0	17.2
3KCo	2009	hd_and_h	*nCNS*	1.8	3.0	27.3	29.4	27.8	27.7	1.53	3.0	19.9	21.1	17.3	18.3
3KYX	2009	all_d	*PHENIX*	1.68	n/a	24.8	26.7	n/a	n/a	1.6	n/a	16.9	19.7	15.9	19.4
3KKX	2009	hd_and_h	*nCNS*	2.0	n/a	27.5	28.6	29.9	32.6	1.5	n/a	16.1	17.3	n/a	n/a
3KYY	2009	hd_and_d	*PHENIX*	1.66	1.09	18.7	20.2	23.9	24.2	1.1	1.35	14.5	15.6	14.2	15.7
3L45	2009	hd_and_h	*nCNS*	1.8	n/a	24.3	30.1	24.5	30.3	1.5	n/a	19.8	21.5	20.1	21.2
3oTJ	2010	hd_and_h	*CNS*	2.15	0	20.9	22.6	20.5	22.4	1.6	n/a	19.8	20.9	18.3	19.7
3QBA	2011	hd_and_d	*nCNS*	1.4	0	30.1	31.5	29.1	29.7	1.53	0	19.4	23.6	19.0	17.6
3QF6	2011	all_d	*PHENIX*	1.85	n/a	n/a	n/a	16.6	21.4	n/a	n/a	n/a	n/a	n/a	n/a
3QZA	2011	hd_and_h	*nCNS*	2.0	2.2	25.4	28.0	25.5	28.2	1.7	2.5	19.5	21.1	17.6	18.7
3R98	2011	hd_and_h	*PHENIX*	2.4	0	20.7	25.1	20.7	25.1	2.1	1.36	16.6	20.3	15.9	19.5
3R99	2011	hd_and_h	*PHENIX*	2.4	0	20.7	25.0	20.7	25.0	2.1	1.36	16.6	20.3	16.0	19.4
3TMJ	2011	hd_and_h	*nCNS*	2.0	n/a	27.6	29.7	27.7	29.8	1.65	n/a	17.5	18.7	17.3	18.2
3VXF	2012	hd_and_h	*PHENIX*	2.75	n/a	18.3	23.4	18.2	24.2	1.6	n/a	16.1	18.4	15.6	18.2
4DVo	2012	hd_and_h	*nCNS*	2.0	2.0	19.0	21.4	19.8	22.2	1.55	2.0	19.4	20.4	17.9	18.7
4GPG	2012	hd_and_h	*PHENIX*	1.98	n/a	19.5	26.0	19.8	25.6	1.9	2.08	14.7	20.3	15.4	20.9
4JEC	2013	hd_and_d	*nCNS*	2.0	3.0	24.4	26.1	25.5	27.4	2.01	n/a	19.4	20.3	18.6	20.5
4LNC	2013	hd_and_h	*PHENIX*	2.19	n/a	28.4	32.2	28.5	31.1	1.84	n/a	15.0	20.9	15.3	19.3
4N3M	2013	hd_and_h	*PHENIX*	1.9	0	24.0	26.7	24.1	26.8	1.92	1.99	14.0	17.2	14.1	17.2
4N9M	2013	hd_and_h	*PHENIX*	2.3	0	25.9	28.8	25.6	28.2	2.02	1.99	16.7	18.9	16.4	18.4
4NY6	2013	hd_and_d	*PHENIX*	1.85	n/a	17.6	22.5	17.6	22.6	1.05	n/a	17.0	18.8	17.0	18.8
3X2o	2014	hd_and_h	*PHENIX*	1.5	n/a	22.8	25.1	22.8	25.1	1.0	n/a	13.5	15.3	13.5	15.2
3X2P	2014	hd_and_h	*PHENIX*	1.52	0	21.8	26.0	220.	26.2	0.99	1.52	13.4	14.2	13.4	14.3
4CVi	2014	hd_and_h	*PHENIX*	2.41	1.58	17.6	24.3	17.9	23.9	2.1	1.37	13.4	17.7	12.9	16.8
4CVJ	2014	hd_and_h	*PHENIX*	2.5	1.34	18.7	27.2	n/a	n/a	2.18	1.38	14.9	20.5	14.4	19.8
4PDJ	2014	hd_and_h	*PHENIX*	1.99	n/a	23.0	27.1	23.0	27.0	1.6	n/a	19.4	21.8	19.4	21.8
4PVM	2014	hd_and_d	*PHENIX*	2.0	1.46	20.9	27.1	20.9	27.3	1.95	1.34	15.3	20.3	15.1	20.0
4PVN	2014	hd_and_d	*PHENIX*	2.3	1.35	20.9	26.2	20.7	25.4	1.95	1.34	15.6	18.5	15.4	18.3
4Q49	2014	hd_and_h	*PHENIX*	1.6	n/a	20.3	21.7	18.2	20.6	1.8	n/a	17.9	18.8	n/a	n/a
4QCD	2014	hd_and_h	*PHENIX*	1.93	n/a	16.7	22.7	17.3	22.8	1.55	n/a	14.3	16.5	14.5	16.6
4QDP	2014	hd_and_h	*nCNS*	2.0	2.5	23.1	24.7	23.7	25.6	1.6	2.5	17.2	18.5	15.5	16.5
4QDW	2014	hd_and_h	*nCNS*	1.8	2.0	16.6	17.9	16.9	18.4	1.6	2.0	18.1	19.0	16.7	17.4
4QXK	2014	hd_and_h	*nCNS*	2.2	2.5	27.7	31.8	26.6	30.4	1.76	n/a	25.7	26.9	25.5	27.6
4S2D	2015	hd_and_h	*nCNS*	2.0	n/a	24.3	27.9	25.5	29.9	1.6	n/a	19.2	19.6	17.9	19.0
4S2F	2015	hd_and_h	*nCNS*	2.0	n/a	26.1	30.4	28.1	32.4	1.7	n/a	19.9	21.1	19.7	21.9
4S2G	2015	hd_and_h	*nCNS*	2.0	n/a	16.4	18.2	17.1	19.8	1.6	n/a	19.6	20.5	18.8	20.5
4S2H	2015	hd_and_h	*nCNS*	1.7	n/a	26.1	26.8	29.2	31.7	1.6	n/a	19.9	21.0	19.0	20.2
4XPV	2015	hd_and_h	*PHENIX*	2.0	n/a	26.4	30.4	26.9	30.7	1.7	n/a	13.3	15.7	13.3	15.8
4Y0J	2015	hd_and_h	*CNS*	2.0	n/a	26.3	29.1	28.7	30.1	n/a	n/a	n/a	n/a	n/a	n/a
5A93	2015	all_d	*PHENIX*	2.2	n/a	21.7	23.6	22.1	23.5	1.6	n/a	13.3	15.6	31.2	31.0
5C6E	2015	hd_and_h	*nCNS*	2.0	2.5	30.1	33.4	29.8	32.5	1.7	2.5	21.0	23.3	19.5	22.0
5C8i	2015	hd_and_h	*nCNS*	2.2	n/a	22.5	27.6	24.6	32.7	1.56	n/a	20.4	22.1	18.8	20.6
5CCD	2015	hd_and_h	*nCNS*	2.6	3.0	20.1	21.4	27.4	32.0	2.2	3.0	20.3	23.9	19.3	22.1
5CCE	2015	hd_and_h	*CNS*	2.5	n/a	34.3	37.6	21.9	25.1	1.82	n/a	25.3	25.7	25.8	27.1
5CE4	2015	all_d	*PHENIX*	1.9	n/a	21.0	25.0	41.5	45.3	0.98	n/a	14.0	16.0	38.6	40.2
5CG5	2015	hd_and_h	*PHENIX*	2.4	n/a	18.6	22.9	18.9	22.7	1.4	n/a	19.4	21.8	20.0	22.4
5CG6	2015	hd_and_h	*PHENIX*	2.4	n/a	26.0	28.7	25.8	25.9	1.7	n/a	19.7	21.1	19.8	20.7
5DPN	2015	hd_and_h	*PHENIX*	1.61	n/a	16.3	20.4	22.3	26.3	1.6	n/a	22.3	25.0	n/a	n/a
5E5J	2015	hd_and_d	*nCNS*	2.0	2.5	21.7	24.5	21.4	24.3	1.85	2.5	19.4	20.1	18.2	19.1
5E5K	2015	hd_and_d	*nCNS*	2.3	n/a	21.2	22.4	24.4	28.9	1.75	n/a	20.3	21.8	19.9	22.0
5EBJ	2015	hd_and_h	*nCNS*	2.5	2.5	30.5	34.4	30.8	35.2	2.1	2.5	23.5	25.3	22.9	24.6
5JPC	2016	hd_and_h	*nCNS*	2.5	n/a	28.2	26.6	31.1	35.4	2.1	n/a	20.8	23.5	21.4	25.2
5JPR	2016	hd_and_h	*PHENIX*	2.2	2.03	23.6	31	n/a	n/a	1.81	1.36	15.5	21.6	15.1	21.5
5K1Z	2016	hd_and_h	*nCNS*	2.6	n/a	25.3	28.7	30.1	37.6	2.25	n/a	20.5	25.8	21.8	27.7
5KWF	2016	hd_and_h	*PHENIX*	2.21	0	28.4	31.2	23.5	26.1	1.5	1.37	19.0	22.0	13.7	16.3
5MoN	2016	hd_and_h	*PHENIX*	1.42	n/a	17.0	18.1	17.0	18.1	0.94	n/a	9.9	10.4	9.9	10.4
5Moo	2016	hd_and_h	*PHENIX*	1.43	n/a	17.0	18.5	17.1	18.4	1.44	n/a	13.4	16.0	13.4	16.0
5T8H	2016	hd_and_d	*nCNS*	2.2	2.5	21.7	25.5	22.1	26.1	1.85	2.5	19.1	21.4	18.1	20.8
5TKi	2016	hd_and_h	*PHENIX*	2.12	n/a	21.6	25.3	21.6	25.1	1.5	0	14.8	17.9	14.7	17.8
5WEY	2017	hd_and_h	*nCNS*	2.5	2.5	24.7	28.5	23.5	27.8	1.8	2.5	19.1	21.2	18.3	21.0

## Footnotes

1 The scattering length of hydrogen is −3.74 fm and that of carbon is +6.65 fm. The sum of the scattering lengths of two H atoms and one C atom is approximately zero.

2 Model 5A93 contains two arrays, but they are identical neutron data arrays.

3 See *File formats and the PDB* (https://www.wwpdb.org/documentation/file-formats-and-the-pdb).

4 Question 2.4 at *wwPDB validation report FAQs* (https://www.wwpdb.org/validation/2016/FAQs): ‘…no reports are currently created for structures determined by neutron diffraction…’.
